# Pembrolizumab in men with heavily treated metastatic castrate‐resistant prostate cancer

**DOI:** 10.1002/cam4.2375

**Published:** 2019-07-03

**Authors:** Matthew D. Tucker, Jason Zhu, Daniele Marin, Rajan T. Gupta, Santosh Gupta, William R. Berry, Sundhar Ramalingam, Tian Zhang, Michael Harrison, Yuan Wu, Patrick Healy, Stacey Lisi, Daniel J. George, Andrew J. Armstrong

**Affiliations:** ^1^ Department of Medicine, School of Medicine Duke University Durham North Carolina; ^2^ Department of Radiology Duke University Durham North Carolina; ^3^ Department of Biostatistics Duke University Durham North Carolina; ^4^ Duke Cancer Institute, Center for Prostate and Urologic Cancers Duke University Durham North Carolina; ^5^ Department of Pharmacy Duke University Hospital Durham North Carolina; ^6^ Department of Pharmacology and Cancer Biology Duke University Durham North Carolina

**Keywords:** genomic profiling, *LRP1b*, mCRPC, pembrolizumab, prostate cancer

## Abstract

**Background:**

Pembrolizumab is approved for patients with metastatic, microsatellite instability (MSI)‐high or mismatch repair‐deficient (dMMR) solid tumors. However, very few men with prostate cancer were included in these initial studies.

**Methods:**

We performed a single institution retrospective review of men with metastatic castrate‐resistant prostate cancer (mCRPC) who were treated with pembrolizumab. The primary objective was to describe the clinical efficacy of pembrolizumab associated with patient and genomic characteristics.

**Results:**

We identified 48 men who received ≥1 cycle of pembrolizumab for mCRPC. Of these, 94% (45/48) had ≥3 prior lines of therapy for mCRPC. Somatic tumor sequencing was available in 18/48 men (38%). We found that 17% (8/48) had a ≥50% confirmed PSA decline with pembrolizumab, and 8% (4/48) had a ≥90% PSA decline with durations of response ranging from 3.1 to 16.3 months. Two of these four men had mutations in *LRP1b*, one of whom also had *MSH2* loss and was MSI‐H and TMB‐high. Despite prior progression on enzalutamide, 48% (23/48) of men were treated with concurrent enzalutamide. The median PSA progression‐free‐survival was 1.8 months (range 0.4‐13.7 months), with 31% of patients remaining on pembrolizumab therapy and 54% of men remain alive with a median follow‐up of 7.1 months.

**Conclusions:**

In a heavily pretreated population of men with mCRPC, pembrolizumab was associated with a ≥50% PSA decline in 17% (8/48) of men, including a dramatic ≥90% PSA response in 8% (4/48), two of whom harbored pathogenic *LRP1b* mutations suggesting that *LRP1b* mutations may enrich for PD‐1 inhibitor responsiveness in prostate cancer.

## 1 INTRODUCTION

Immune checkpoint inhibitors (ICI) have dramatically altered the treatment paradigm for numerous solid tumors including melanoma, lung, kidney, and bladder cancer.^1^, ^2^, ^3^, ^4^ Unfortunately, initial studies designed to investigate the potential for ICI in metastatic castrate‐resistant prostate cancer (mCRPC) were discouraging. A phase I study of nivolumab in 17 unselected patients with mCRPC demonstrated an overall response rate of 0% (0/17).^5^ Two phase 3 studies showed no survival improvement in unselected patients treated with CTLA‐4 inhibition.^6^, ^7^ However, more recent studies have begun to show responses in select patients. In 2016, Graff et al showed early evidence of anti‐PD‐1 activity, using pembrolizumab plus enzalutamide, in 3 out of 10 patients who had previously progressed after treatment with enzalutamide alone based on striking PSA declines and radiographic responses which were durable.^8^ These data were updated and suggest an 18% PSA response rate in genomically unselected men.^9^

In May 2017, the US Food and Drug Administration (FDA) granted accelerated approval to pembrolizumab for patients with unresectable or metastatic, microsatellite instable‐high (MSI‐H) or mismatch repair‐deficient (dMMR) solid tumors that have progressed on prior therapy and have no satisfactory treatment options. This was the first drug authorized for use based on a molecular biomarker rather than a traditional histopathologic diagnosis.^10^ Of the 149 patients in the studies the approval was based from, two had prostate cancer, one with a partial response, and one with stable disease.^11^ MSI‐high status has been reported in the range of 2.2%‐12% of patients with advanced prostate cancer.^12^, ^13^ For this reason, current NCCN guidelines for the treatment of prostate cancer recommend MSI testing for men with mCRPC and pembrolizumab therapy for those men with refractory MSI‐high mCRPC.^14^

Recent studies support the efficacy of PD‐1 inhibition in select patients with mCRPC. One reported 80% (4/5) of patients with MSI‐high mCRPC treated with either nivolumab or pembrolizumab had PSA reductions of more than 50%.^15^ In unselected patients, only 11% of men with mCRPC had 50% or greater PSA declines with pembrolizumab and only 3%‐4% of men had objective radiographic responses in the phase 2 KEYNOTE‐199 study of pembrolizumab monotherapy.^16^ A recent study of over 1000 men with mCRPC and tumor samples adequate to undergo MSI‐sensor testing found that 2.2% of samples had high MSI‐sensor scores and an additional 9% with indeterminate scores with evidence of dMMR. Eleven patients with MSI‐H/dMMR mCRPC received anti–PD‐1/PD‐L1 therapy. Six of these men (54.5%) had a ≥50% decline in PSA levels, four of whom had radiographic responses.^12^ Together, these data support the clinical activity of PD‐1 blockade in selected men with mCRPC and suggest that subsets of men with MSI‐high and those with concurrent therapy with enzalutamide may have greater activity.

Along with MSI, dMMR, and potentially PD‐L1, another biomarker that has recently gained interest for potential immunogenicity is *CDK12*.^17^ Wu et al performed integrative genomic analyses on 360 samples from men with mCRPC and identified a novel immunogenic subtype typified by biallelic loss of *CDK12*, elevated neoantigen burden, increased T‐cell infiltration and clonal expansion, and heightened response to anti‐PD1 monotherapy, with 50% (2/4) having marked PSA reductions.^17^

Our study sought to retrospectively review all men who were treated at the Duke Cancer Center with pembrolizumab for the treatment mCRPC and to define whether the clinical benefits of pembrolizumab could be predicted by specific genomic alterations identified by FoundationOne molecular profiling.

## 2 METHODS

### 2.1 Patients and eligibility

We performed a retrospective review of all men with mCRPC treated at the Duke Cancer Center who received pembrolizumab between January 1, 2013 and March 1, 2018. All patients were required to be ≥18 years of age and have biopsy proven adenocarcinoma of the prostate, progression of disease after standard of care androgen deprivation therapy or subsequent anti‐neoplastic therapy, metastatic disease based on imaging, and have received at least one cycle of pembrolizumab for the treatment of mCRPC. Any patients previously treated with a checkpoint‐inhibitor and any patients receiving pembrolizumab as part of a clinical trial were excluded. IRB approval for this consent‐exempt chart review was obtained prior to review of the electronic medical records used to abstract clinical data. Clinical data (including pathologic and laboratory data) were recoded and secured in a password‐protected, auditable, IRB‐approved REDCap database.

### 2.2 Pembrolizumab treatment

A total of 58 men were identified who received at least one dose of pembrolizumab for the treatment of mCRPC. Ten received treatment as part of a clinical trial and were excluded from further analysis. Of the remaining 48 men, all received pembrolizumab 200 mg infused intravenously over 30 minutes once every 3 weeks per cycle. Only two men had insurance approval for pembrolizumab. The other 46 all received pembrolizumab without cost via the Merck expanded access program with assistance from Duke Oncology specialty pharmacists. Enzalutamide was dosed per standard of care and continued with pembrolizumab in men with disease progression on prior enzalutamide therapy.

### 2.3 Outcomes

We quantified PSA declines from baseline with pembrolizumab and before subsequent therapy and required confirmation with a second value at least 2 weeks apart. Concurrent therapy with enzalutamide or abiraterone was allowed if the patient had prior documented PSA progression on that treatment. PSA was measured on day 1 of each cycle, and the median interval between PSA testing was 21 days (range 6‐101). Duration of therapy was defined as the time of treatment initiation until treatment discontinuation for any reason. PSA‐PFS was defined as time from treatment initiation until first PSA value at a 25% increase from baseline total serum PSA confirmed by repeat value at least two weeks apart.

Board‐certified, fellowship‐trained radiologists with expertise in abdominopelvic imaging and RECIST calculations (DM and RTG) reviewed all available imaging to assess for radiographic progression free survival as well as best overall objective response per RECIST 1.1^18^ and PCWG3^19^ criteria. Imaging was typically performed every 2‐3 months while on therapy but was not mandated given the clinical nature of this compassionate use program for pembrolizumab.

### 2.4 Genomic profiling

All genomic profiling included in this review was obtained utilizing standard‐of‐care FoundationOne next‐generation sequencing of archival somatic tumor samples (either from the original prostate biopsy or from sites of metastases.) Source tissue was abstracted from FoundationOne reports and recorded for each patient.

### 2.5 Statistical analysis and objectives

The primary objective of this analysis was to describe the observed efficacy of pembrolizumab in men with mCRPC as described by confirmed PSA declines from baseline, time to treatment discontinuation, radiographic responses, and overall survival. The secondary objectives were to describe the specific genomic results of all patients who received FoundationOne testing, including the microsatellite instability status, the degree of tumor mutational burden, and the presence of *CDK12* and other mutations when available according to response status. For patients with degree of tumor mutational burden (TMB) in mutations per megabase (muts/Mb) available, patients were divided into TMB low (≤5 muts/Mb), TMB intermediate (6‐19 muts/Mb), and TMB high (≥20 muts/Mb).^20^ No formal sample size calculation was necessary as this was a descriptive retrospective analysis and all patients who met inclusion criteria were included.

## 3 RESULTS

**Figure 1:**
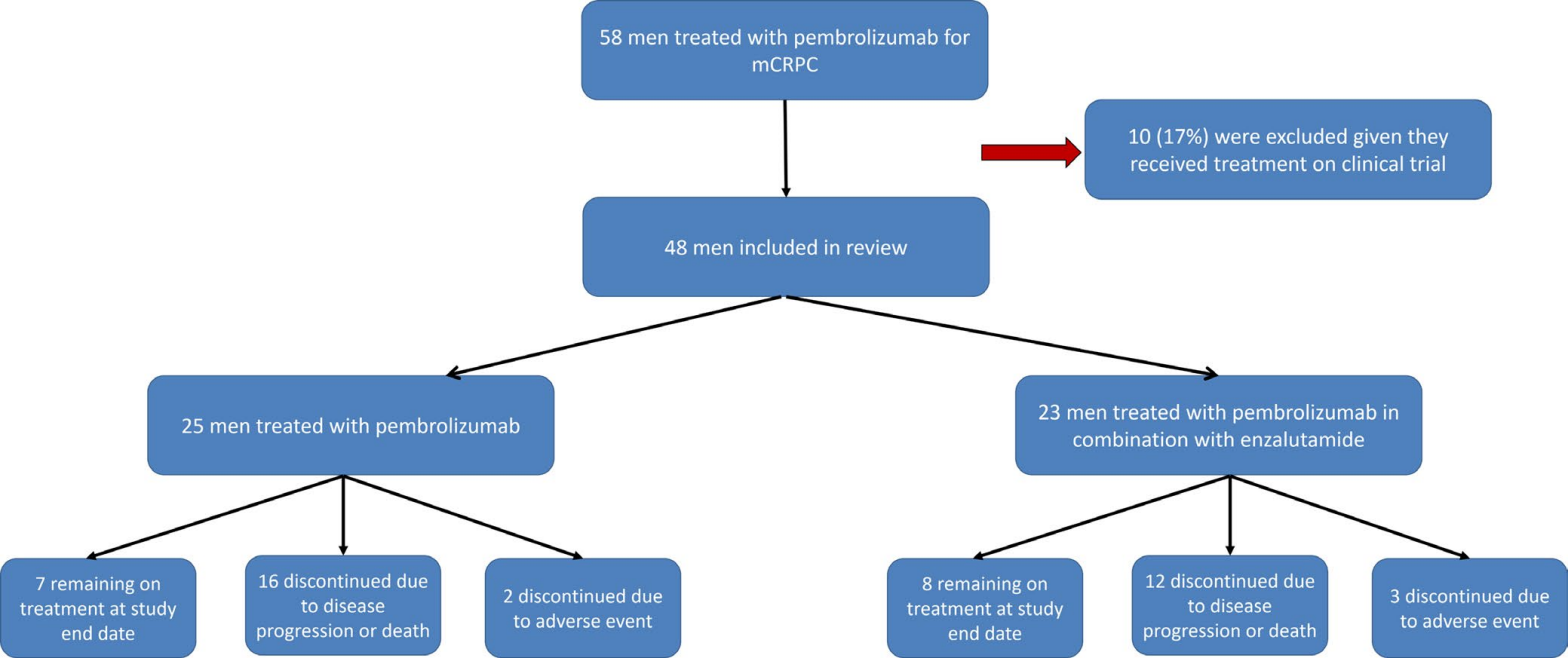
CONSORT diagram

A total of 48 men who received ≥1 cycle of pembrolizumab for mCRPC were included for review; baseline characteristics including prior therapy are available in Table 1. The median baseline PSA was 117.7 ng/ml. Overall, 94% (45/48) had received three or more prior lines of therapy after ADT, including docetaxel (90%), abiraterone (88%), enzalutamide (85%), and sipuleucel‐T (73%). Fifty‐two percent (25/48) of men were treated with concurrent therapy along with pembrolizumab, most commonly enzalutamide (48%, 23/48) despite prior progression on this therapy. Additionally, 54% (26/48) of men had visceral metastatic disease, most commonly hepatic (33%) and pulmonary (19%) metastases. See CONSORT diagram for patient inclusion and characteristics (Figure 1). The median number of pembrolizumab 3‐week cycles was four cycles (range 1‐18 cycles) and 19% (9/48) of men received >6 months of pembrolizumab therapy.

**Table 1:** Baseline characteristics

	All patients (48)	Pembrolizumab (25)	Pembrolizumab and enzalutamide (23)
Age, median (range), years	73 (51‐87)	74 (51‐87)	70 (59‐83)
Caucasian (%)	43 (90)	24 (96)	19 (82)
African American (%)	4 (8)	1 (4)	3 (13)
Asian (%)	1 (2)	0 (0)	1 (4)
Pattern of Spread			
Node only	3 (6)	1 (4)	2 (9)
Bone	43 (90)	23 (92)	20 (87)
Visceral	26 (54)	14 (56)	12 (52)
Hepatic	16 (33)	8 (32)	8 (35)
Pulmonary	9 (19)	5 (20)	4 (17)
Other	9 (19)	3 (12)	6 (26)
ECOG 0‐1 (%)	36 (75)	16 (64)	19 (83)
Gleason >8 (%)	22/40 (55)	7/18 (39)	15/22 (68)
Median PSA ng/ml (range)	117.68 (6.68‐7595)	98.3 (6.68‐4732)	133.0 (8.02‐7595)
Median LDH U/L	197	199	194
Median Albumin g/dl	3.6	3.5	3.6
Median Hemoglobin	10.6	10.9	10.5
Previous Treatment (%)			
Docetaxel	43 (90)	22 (88)	21 (91)
Abiraterone	42 (88)	23 (92)	19 (83)
Enzalutamide	41 (85)	20 (80)	21 (91)
Sipuleucel‐T	35 (73)	21 (84)	14 (61)
Cabazitaxel	23(48)	14 (56)	9 (39)
Carboplatin	16 (33)	9 (36)	7 (30)
Radium‐223	14 (29)	7 (28)	7 (30)
Olaparib	2 (4)	1 (4)	1 (4)

### 3.1 Efficacy

Overall, 21% (10/48) of men had a ≥30% confirmed PSA decline, 17% (8/48) had a ≥50% confirmed PSA decline, and 8% (4/48) had ≥90% confirmed PSA decline with pembrolizumab. Of the 23 men treated with pembrolizumab/enzalutamide after progression on enzalutamide, 5 (22%) had a confirmed ≥50% PSA decline. All of these men had documented PSA progression while previously on enzalutamide. The proportion of men with any PSA decline with pembrolizumab alone or with pembrolizumab plus enzalutamide was 40% (10/25) and 48% (11/23), respectively. Six of the eight men with confirmed ≥50% PSA decline had evaluable CT imaging at least 3 months out from their first dose of pembrolizumab. Among those men with a ≥50% confirmed PSA decline, we observed one complete response (CR), two partial responses (PR), two stable disease (SD), and one with progressive disease (PD) by RECIST 1.1 (Table 2). There were no confirmed imaging responses in men without PSA response per investigator review, and thus, the overall objective response rate was 6.3% (3/48), 12% in men with visceral metastases (3/26).

**Table 2:** Summary efficacy table

	All patients (48)		Pembrolizumab only (25)		Pembrolizumab and enzalutamide (23)	
≥30% PSA decline	(10/48)	20.8%	(5/25)	20.0%	(5/23)	21.7%
≥50% PSA decline	(8/48)	16.7%	(3/25)	12.0%	(5/23)	21.7%
≥90% PSA decline	(4/48)	8.3%	(1/25)	4.0%	(3/23)	13.0%
Median PSA‐PFS (range)	55 (13‐411)		36 (13‐411)		63 (21‐162)	
Patients without PSA progression at study end date, %	(7/48)	14.6%	(2/25)	8.0%	(5/23)	21.7%
≥3 months without PSA progression	(18/45)	40.0%	(6/23)	26.1%	(12/22)	54.6%
≥6 months without PSA progression	(7/42)	16.7%	(4/22)	18.2%	(3/20)	15.0%
Patients Still on treatment at end of study, %	15 (31)	31.3%	(7/25)	28.0%	(8/23)	34.8%
Median OS (range)	Not met		199, (21‐)		Not met	
Patients alive at study end date, %	26 (54)	54.2%	(11/25)	44.0%	(15/23)	65.2%

Summary efficacy table highlighting percentage of men with confirmed ≥30%, 50%, and 90% decline in total serum PSA values, median PSA‐Progression free survival, and median overall survival.

Of the 45 men who had at least 3 months follow‐up, 40% (18/45) had a PSA PFS of at least 3 months, and of the 42 men who had at least 6 months follow‐up, 17% (7/42) had a PSA PFS of at least 6 months. The median time to discontinuation of treatment 5.2 months. Overall, the median PSA PFS was 1.8 months (range 0.4‐13.7), 1.2 months (range 0.4‐13.7) among the 25 men treated with pembrolizumab alone, and 2.1 months (range 0.7‐5.4) among the 23 men treated with combination pembrolizumab and enzalutamide. The median overall survival was not met in 54% of men alive at the study end point with a median follow‐up time of men alive at the study end point of 7.1 months. Among the eight men with a ≥50% PSA decline, duration of response ranged from 3.1 to 16.3 months with four of the eight men having ongoing responses at the study end date (median follow‐up 6.4 months). Table 2 shows summary efficacy data and Figure 3 shows corresponding PSA waterfall graph and patient spider plot.

### 3.2 PSA kinetics

There were 35 men with both three pretreatment PSA values and three posttreatment PSA values available for PSA kinetics calculations. Of these, the eight men with ≥50% PSA response were excluded in order to prevent a skewed response. Using linear regression, the remaining 27 men had a mean slope of the pre‐pembrolizumab PSA values of 2.23 ng/ml/day (range −4.33 to 20.47), mean slope of the post‐pembrolizumab PSA values of 2.00 ng/ml/day (range −11.49 to 16.15), and a mean difference in slope of −0.23 ng/ml/day (range −7.15 to −4.32). Of these 27 men, 8 had a negative difference in slope indicating a reduction in PSA velocity while on treatment.

### 3.3 Genomic profiling

**Figure 2:**
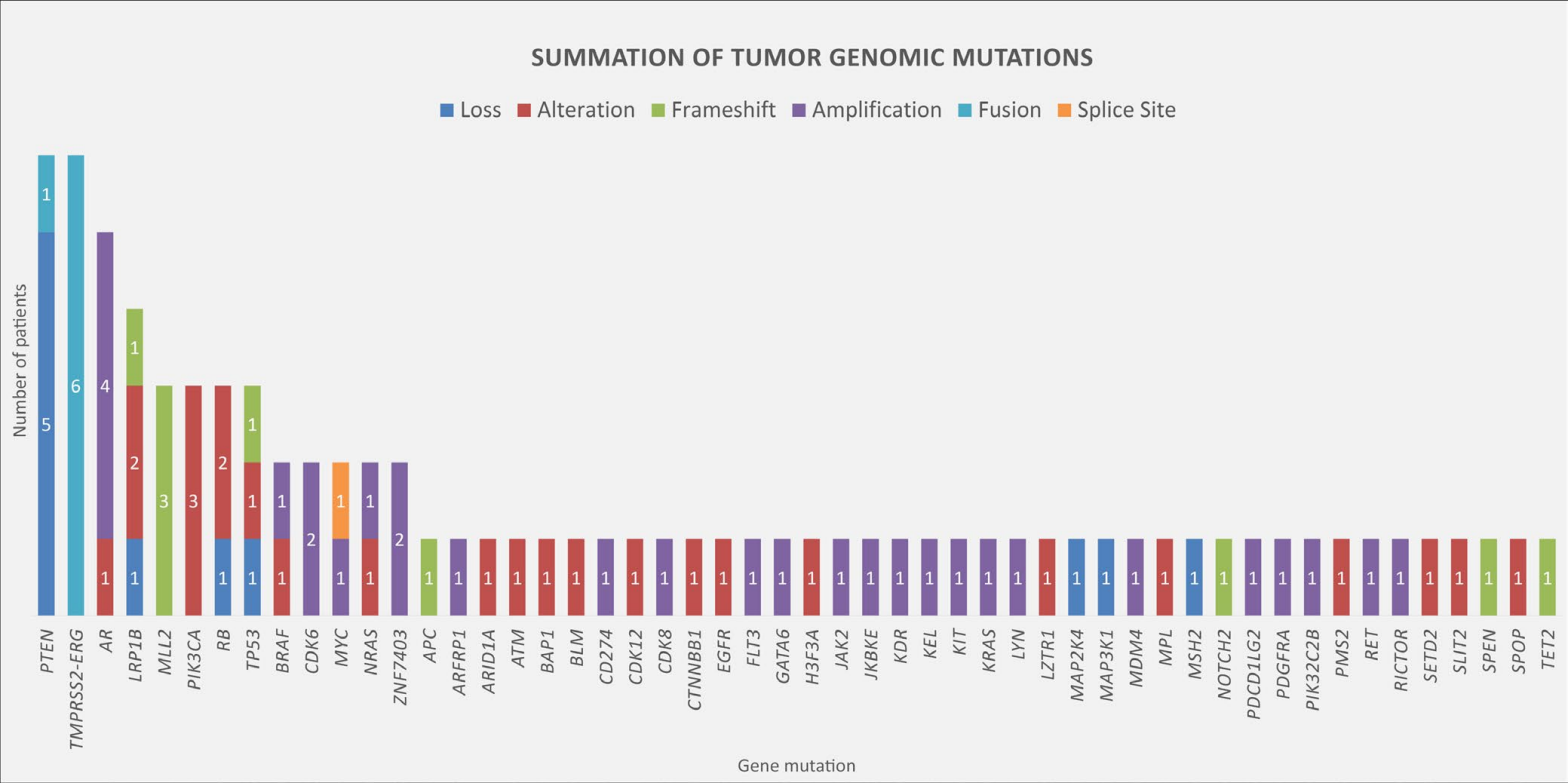
Summation of tumor genomic mutations. Representation of all reported mutations present from all 18 men with available FoundationOne profiling. Four *LRP1b* mutations present: one loss, two missense mutations, and one frameshift mutation

Somatic tumor sequencing via FoundationOne was available and had evaluable results in 18/48 men (38%). Six men had testing from their primary prostate biopsy and/or surgical prostatectomy specimen, while the remaining 12 had testing from metastatic sites. The most frequently reported alterations were the *TMPRSS2‐ERG* fusion 33% (6/18), *PTEN* loss 28% (5/18), and *AR* amplification 22% (4/18) (Figure 2 **)**. Only 1 of the 18 harbored a mutation in *CDK12* (6%), and only 1 (6%) was found to be MSI‐high. The one patient who was MSI‐high also had high TMB and also had a pathogenic mutation present in the low‐density lipoprotein receptor‐related protein 1b (*LRP1b*). Of the four men with confirmed ≥90% PSA reductions, two had molecular profiling available and the other two did not. Both men with profiling present harbored mutations in *LRP1b*, one of whom was the patient who was both MSI‐high and TMB‐high **(**Table 3 **).** Overall, the prevalence of *LRP1b* mutations in our cohort was 22% (4/18), and the response to pembrolizumab (PSA decline ≥50%) in this genomic subset was 75% (3/4, Table 3), with duration of response ranging from 4.6 to 16.3 months. Among men with genomic profiling not revealing mutations in *LRP1b*, the response to pembrolizumab (PSA decline ≥50%) was 14% (2/14), with duration of response ranging from 0.7 to 4.2 months.

**Figure 3:**
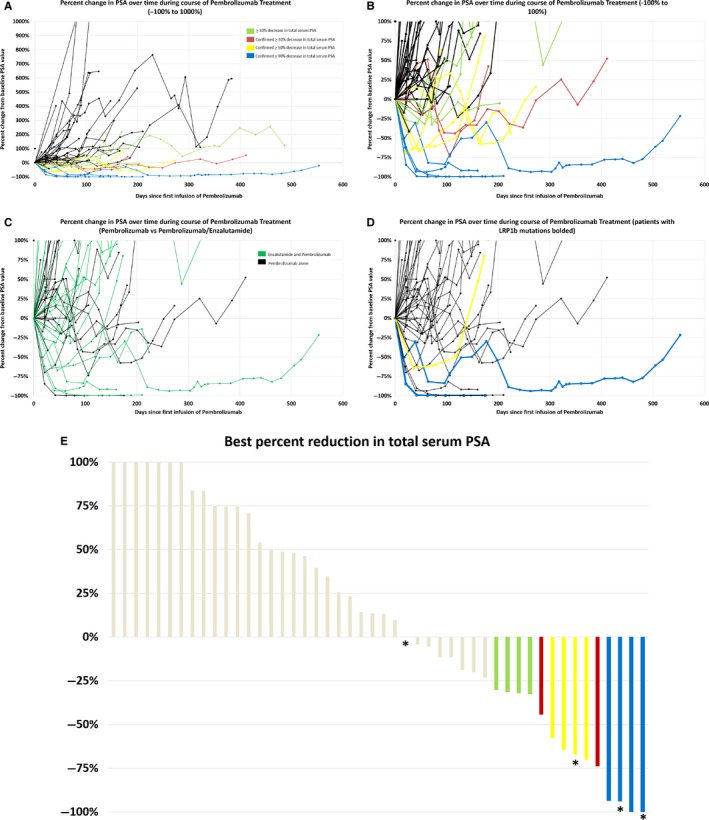
Percent change in PSA over time on pembrolizumab. (A‐D) Spider plot showing percent change in total serum PSA levels over time (days) on treatment with pembrolizumab. (A) −100% to 1000 change. (B) −100% to 100% change. (C) Patient treated with combination enzalutamide and pembrolizumab highlighted in green. (D) Patients harboring *LRP1b* mutations heighted in yellow for ≥50% decrease in PSA and blue for ≥90% decrease in PSA. (E) Waterfall plot showing best percent reduction in total serum PSA. Asterisk indicates presence of LRP1b mutation

**Table 3:** Characteristics of patients with confirmed ≥50% PSA decline

Patient ID	Foundation One available	LRP1b alteration present	MSI‐status	TMB‐status	Best PSA response	Duration of response	Best imaging response	Lines of treatment beyond ADT	Concurrent treatment
1	Yes	Yes	MSI‐H	High; 29 Muts/Mb	−99%	5.5^a^	CR	2	None
2	Yes	Yes	Stable	Itm; 6.39 Muts/Mb	−94%	16.3	SD	5	Enzalutamide^b^, abiraterone^b^
3	No	n/a	n/a	n/a	−99%	6.6^a^	PR	6	Enzalutamide^b^
4	No	n/a	n/a	n/a	−67%	6^a^	n/a	6	Enzalutamide^b^
5	No	n/a	n/a	n/a	−70%	3.7	PD	7	None until progression
6	Yes	No	Stable	Low; 4 Muts/Mb	−58%	3.1^a^	PR	4	None
7	No	n/a	n/a	n/a	−94%	4.7^a^	n/a	6	Enzalutamide^b^
8	Yes	Yes	Stable	n/a	−64%	4.6	SD	6	Enzalutamide^b^

Characteristics of the eight men with confirmed ≥50% declines in total serum PSA. 75% (3/4) harbored mutations in *LRP1b*. One patient had a complete imaging response and two had partial imaging responses. Four patients had MSI‐status available and only one was MSI‐high.

a Response ongoing at study end date.

b Previously progressed on concurrent treatment.

**Figure 4:**
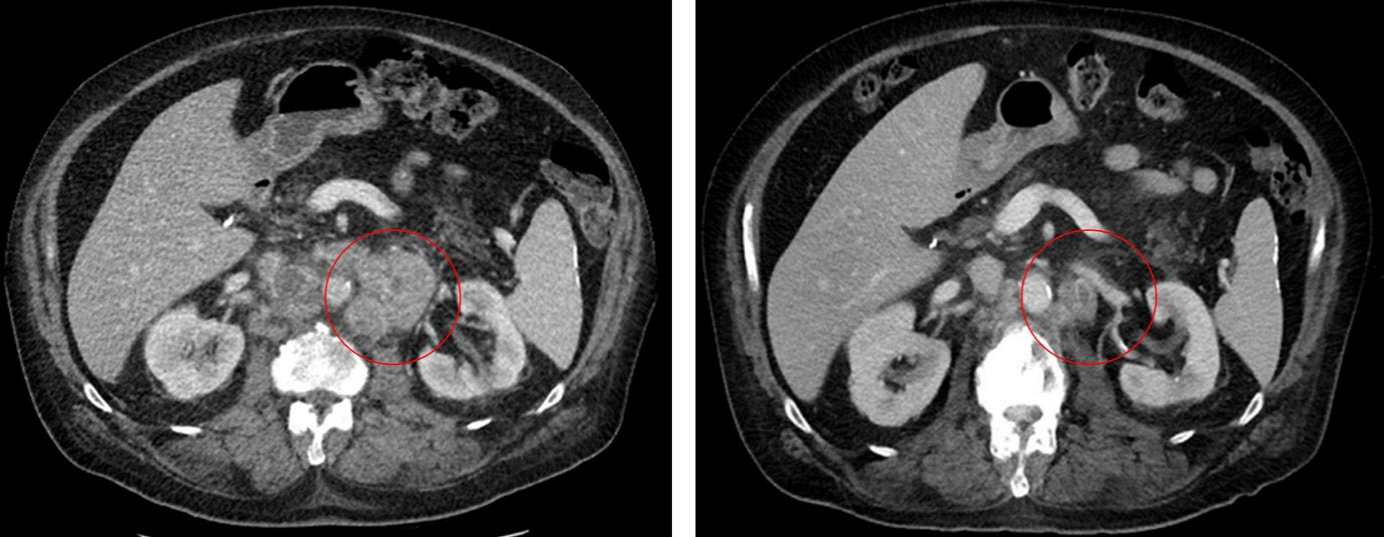
Radiograph showing partial response from patient 3. Representative CT scans of a patient with a partial imaging response (PR). CT scan 3 months out (right) shows substantially decreased size of bulky retroperitoneal lymphadenopathy. His PSA remained >90% below baseline and he remained on treatment at study end date, having received 11 cycles thus far

Detailed descriptions, including age, race, initial Gleason score, and complete genomic alterations, of individual responding patients and key molecular subsets of men with mCRPC are detailed in supplements. Briefly, patient 1’s PSA declined from 44.3 ng/ml to a nadir of 0.10 ng/ml, eventually having complete imaging response; his genomic profiling revealed somatic *MSH2* loss as well as gene alterations in *EGFR*, *NRAS*, *BAP1*, *LRP1b* (T592fs*4), *MLL2*, and *SPEN* along with MSI‐High and TMB‐High (29 Muts/Mb). Patient 2’s PSA substantially decreased from 2,971 ng/ml to a nadir of 183 ng/ml, and he completed 18 cycles of pembrolizumab prior developing a presumed immune mediated pancreatitis; his genomic profiling revealed alterations in *AR*, *PTEN*, *LRP1b* (E4581*), and *SUPT3H‐PTEN* fusion along with TMB‐intermediate (6.39 Muts/Mb) and MS‐stable. Patient 3’s PSA declined substantially from 1048 ng/ml to a nadir of 2.55 ng/ml. Imaging approximately three and a half months out revealed a partial response with decreased size of bulky retroperitoneal lymphadenopathy and retrocrural lymphadenopathy (Figure 4.) Patient 4’s PSA declined rapidly from 63.66 ng/ml to a nadir of 3.96 ng/ml within the first 3 months of therapy. Neither patient 3 nor 4 had genomic profiling available for review. Patient 8 had a mutation in *LRP1b* (*R1815W* reported as a variable of undetermined significance) in addition to an *ATM* (Q2414*) mutation. His PSA declined from 16.25 ng/ml to a nadir of 5.78 ng/ml with CT scan about two months into treatment showing stable disease. Of the remaining patients with genomic profiling available, only one other patient had a mutation in *LRP1b* (*LRP1b* loss of exons 4‐91). However, no post‐treatment PSA levels were available given that he transitioned to hospice following a hospitalization soon after his first infusion. One patient had alterations in genes both for PD‐L1 and for PD‐L2, *CD274* amplification and *PDCD1LG2* amplification respectively, but he did not have evidence of confirmed response. Only one patient had a mutation in *CDK12* (*CDK12* splice site 2666+1G>T), and he also did not have evidence of response.

### 3.4 Toxicity

Approximately 35% (17/48) of men treated had a documented adverse event. Six men (13%) received steroids for treatment of presumed immune‐mediated adverse events, and treatment was eventually discontinued in seven men secondary to adverse events. There were 5 grade 1 events, 13 grade 2 events, 3 grade 3 events, and no grade 4‐5 events (Table 4).

**Table 4:** Toxicity after initiation of pembrolizumab

Adverse events	Grade (number of subjects)
Endocrine disorders: hypothyroidism	2 (4)
Pulmonary disorders: pleural effusion	3 (1)
GI disorders:	
Nausea	2 (2)
Diarrhea	1 (3)
Pancreatitis	2 (1)
Skin and subcutaneous tissue disorders: maculopapular rash	1 (1)
Musculoskeletal and connective tissue disorders:	
Arthralgias	1 (1), 2(2)
Myalgias	3 (1)
Nervous system Disorders:	
Confusion	2 (1)
Vison change	2 (1)
Fatigue	3 (1)
Personality change	2 (1)
Eye disorders: dry eyes	1 (1)

## 4 DISCUSSION

In this retrospective study of pembrolizumab therapy for men with mCRPC, we identified several clinically important findings. First, we identified confirmed 30% or greater PSA declines in 21% (10/48) of patients overall, with 12% (3/26) of men with visceral disease having confirmed partial or complete objective imaging responses. Second, we report a 22% (5/23) confirmed PSA response rate (≥50% decline) in men with mCRPC treated with combination pembrolizumab and enzalutamide, despite prior progression on enzalutamide. Third, of the 38% of patients with evaluable genomic profiling, we found several significant genomic alterations present in responding men, particularly MSI high status and *LRP1b* mutations. Only one patient each had alterations in PD‐L1/PD‐L2 and *CDK12*, and neither had evidence of response.

**Figure 5:**
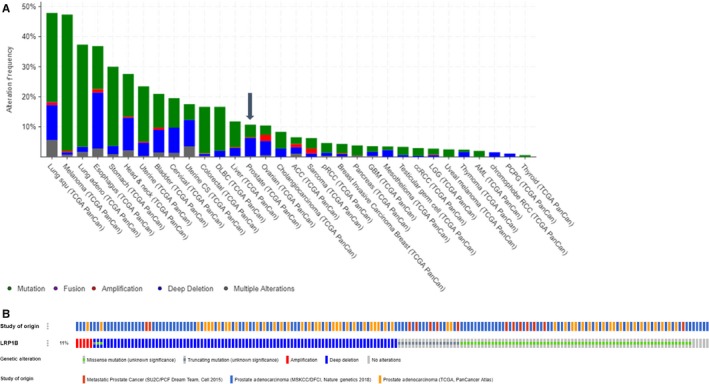
(A) LRP1b across all cancer types as per cBIOPORTAL. (B) LRP1b alterations present in prostate cancer as per cBIOPORTAL. (A) TCGA data from cBIOPORTAL showing prevalence of *LRP1b* mutations across tumor types; approximately 11% prevalence in prostate cancer. B) Prevalence of *LRP1b* mutations is from TCGA (yellow) approximately 11% (53/494), 11% MSKCC/DFCI (111/1013), and SU2C/PCF (red) 9% (14/150)^26^, ^27^, ^28^

Of the four patients with ≥90% PSA reduction two had alterations in *LRP1b*, one of whom was also MSI‐high and TMB‐high. *LRP1b* is a large gene approximately 500 kilobases on the long arm of chromosome 2.^21^ Molecular studies have demonstrated *LRP1b* expression in patients with lung, urothelial, and cervical carcinomas.^22^, ^23^, ^24^ Figure 5A shows the prevalence of *LRP1b* mutations across cancer types based on data from The Cancer Genome Atlas (TCGA) data available on the cBIOportal database,^25^, ^26^, ^27^ and Figure 5B highlights data from TCGA,^27^ Memorial Sloan Kettering Cancer Center/Dana‐Farber Cancer Institute (MSKCC/DFCI),^28^ and Stand Up to Cancer‐Prostate Cancer Foundation (SU2C/PCF)^29^ that together reveal a combined prevalence *LRP1b* mutations in approximately 11% of men (Figure 5B). Additionally, a recent study using four computational tools evaluated men with prostate cancer from the TCGA and found that *LRP1b* was one of the top 10 most frequent driver mutations*,* present in approximately 3.9% of tumors.^30^ In our heavily treated cohort, 22% (4/18) of the men with genomic profiling available had *LRP1b* loss or mutation present.

In a retrospective review of patients with metastatic melanoma treated with ICI, *LRP1b* mutation was found to significantly correlate with response, being present in 11/32 (34%) of responders compared to 1/33 (3%) in non‐responders (*P* < 0.001).^31^ The authors hypothesized that, given *LRP1b's* size and genomic instability, it may serve as a single‐gene surrogate for total mutational load, having also found *LRP1b*‐mutated patients to have a significantly higher mutational load compared to those without. Of our four patients with *LRP1b* alterations, one was MTB high (and MSI‐high), one was MTB intermediate, and the other two were TMB unknown. A retrospective review of 190 patients with metastatic nasopharyngeal carcinoma who underwent genomic profiling also showed a significant correlation between *LRP1b* and TMB‐high status.^32^ An impressive 75% (3/4) PSA response rate among men with *LRP1b* mutations was observed in this study, and additional studies are necessary to further elucidate the relationship between *LRP1b* and response to ICI.

There are several limitations to this study. First, this was a retrospective review of all mCRPC patients at an academic referral center who were selected to receive pembrolizumab, and thus our experience may not reflect that activity in a broader community‐based or prospective study. Second, tumor genomic profiling was only available in 38% of patients, and when profiling was available, microsatellite status and TMB profile were not always included at the time of FoundationOne testing during the period of this chart review. However, these data are now routinely provided on tissue genomic profiling, and somatic and germline molecular profiling of all men with metastatic prostate cancer is now routinely performed at our institution. Third, almost half of the men were concomitantly treated with enzalutamide in addition to pembrolizumab (Figure 3C). While these men already had PSA progression while previously being treated with enzalutamide, it is unknown if this addition may have impacted the tumor microenvironment. For example, enzalutamide may induce PD‐L1 expression in prostate cancer cells and in dendritic cells, supporting the concept that concurrent therapy with PD‐1 blockade and enzalutamide may have synergy.^33^ The ongoing phase 3 trial of enzalutamide with or without pembrolizumab (NCT03834493) will test the clinical benefits of this combination prospectively. Finally, the major limitation of this study is our small sample size. While this cohort represents one of the largest published descriptions of pembrolizumab activity in men with mCRPC, observing genomic alterations of significance and correlating with response requires large samples sizes to help elucidate rare predictive biomarkers.

## 5 CONCLUSION

Our results demonstrate that pembrolizumab may be an effective therapy for a minority of men with advanced prostate cancer, with 17% of men having ≥50% reduction in total serum PSA and 8% with >90% reduction. Among men without ≥50% PSA decline, eight men still had a negative difference in their post‐treatment PSA slope, indicating a reduction in PSA velocity while on treatment. Additionally, 40% of men were without PSA‐progression for ≥3 months and 17% without PSA‐progression for ≥6 months. While currently only FDA approved for men with MSI‐H or dMMR status,^9^ our study highlights *LRP1b* as another potential marker for response along with the degree of TMB. More biomarker driven studies are needed to further elicit which patients have the greatest potential for response.

## CONFLICT OF INTEREST

Jason Zhu reports personal fees from Bayer, outside the submitted work. Tian Zhang reports grants from Acerta, Novartis, Merrimack, Abbvie/Stemcentrx, Merck, Regeneron, Exelixis, Janssen, Pfizer, OmniSeq, and Personal Genome Diagnostics; services on speaker's bureau and advisory boards for Genentech/Roche, Exelixis, Sanofi‐Aventis; services on advisory board for Janssen, Astra Zeneca, Pfizer, Amgen, Bristol Myers Squibb (BMS); consultant services for Bayer, AstraZeneca, and Foundation Medicine; spouse is an employee and stockholder for Capio Biosciences all outside the submitted work. Michael Harrison reports grants from Merck, Genentech, AstraZeneca, Pfizer, BMS, and Janssen; personal feeds from Bayer, Genentech, AstraZeneca, Pfizer, BMS, and Janssen, all outside the submitted work. Daniel J. George reports grants from Janssen, Astellas/Pfizer, Dendreon, Bayer, Novartis, BMS, and Genentech/Roche; personal fees from Janssen, Astellas/Pfizer, Bayer, Merck, BMS, and Genetech/Roche; consulting services for Janssen, Astellas/Pfizer, Bayer, Merck, BMS, and Genentech/Roche, all outside the submitted work. Andrew J. Armstrong reports grants from Janssen, Astellas/Pfizer, Dendreon/San Power, Bayer, Novartis, Merck, BMS, Genetch/Roche, Active Biotech, Constellation, and AstraZenica; personal fees from Janssen, Astellas/Pfizer, Dendreon/San Power, Bayer, and Merck; consulting services for Janssen, Astellas/Pfizer, Dendreon/San Power, Bayer, Merck, and AstraZenica, all outside the submitted work. Matthew D. Tucker, Daniele Marin, Rajan T. Gupta, Santosh Gupta, William R. Berry, Sundhar Ramalingam, Yuan Wu, Patrick Healy, and Stacey Lisi collectively have no disclosures to report.

## AUTHOR CONTRIBUTION

Matthew D. Tucker: Data collection and analysis, writing initial draft, critical revision of the article for important intellectual content, and approval of the final version. Jason Zhu: Data collection and analysis, writing initial draft, critical revision of the article for important intellectual content, and approval of the final version. Daniele Marin: Formal RECIST calculations of available imaging and approval of the final version. Rajan T. Gupta: Formal RECIST calculations of available imaging and approval of the final version. Santosh Gupta: Critical revision of the article for important intellectual content, and approval of the final version. William R. Berry: Critical revision of the article for important intellectual content, and approval of the final version. Sundhar Ramalingam: Critical revision of the article for important intellectual content, and approval of the final version. Tian Zhang: Critical revision of the article for important intellectual content, and approval of the final version. Michael Harrison: Critical revision of the article for important intellectual content, and approval of the final version. Yuan Wu: Critical revision of the article for important intellectual content, and approval of the final version. Patrick Healy: Critical revision of the article for important intellectual content, and approval of the final version. Stacey Lisi: Critical revision of the article for important intellectual content, and approval of the final version. Daniel J. George: Critical revision of the article for important intellectual content, and approval of the final version. Andrew J. Armstrong: Study concept and design, data collection and analysis, critical revision of the article for important intellectual content, and approval of the final version.
