# Supplementary material for: Pembrolizumab in men with heavily treated metastatic castrate‐resistant prostate cancer
Source: Cancer Med. 2019 Jul 3;8(10):4644–55. doi: 10.1002/cam4.2375 (PMC6712455; doi:10.1002/cam4.2375)
Supplement: Supplementary file 1 [file CAM4-8-4644-s001.docx]

*Supplemental Clinical Vignettes of Responding Patients*

Patient 1 is a 76-year-old Caucasian male, with initial Gleason 3+4, who previously progressed on enzalutamide and sipuleucel-T for mCRPC and underwent FoundationOne genomic profiling from tissue collected during surgery for local invasion into the bladder causing bladder outlet obstruction. Molecular profiling revealed somatic *MSH2* loss as well as gene alterations in *EGFR*, *NRAS*, *BAP1*, *LRP1b* (*T592fs*4*), *MLL2*, and *SPEN* and his tumor was also found to be MSI-High and TMB-High (29 Muts/Mb). He was subsequently started on pembrolizumab monotherapy. His PSA declined rapidly from 44.3 ng/mL to a nadir of 0.10 ng/mL within the first three months of therapy and imaging after three months of therapy revealed a partial response with significantly decreased size of retroperitoneal and pelvic lymphadenopathy. His response was maintained, eventually leading to a complete imaging response. He was still on treatment at the study end date, having received 5 cycles (15 weeks) of pembrolizumab.

Patient 2 is a 59-year-old Caucasian male, with initial Gleason 4+3 , who developed disease progression after abiraterone, enzalutamide, sipuleucel-T, radium-223, and docetaxel for mCRPC, and developed a significant burden of disease on imaging with diffuse osseous metastases, peritoneal carcinomatosis, and ascites prior to starting pembrolizumab. Somatic FoundationOne profiling of a prostate biopsy around the time of initiation of pembrolizumab revealed alterations in *AR*, *PTEN*, *LRP1b* (*E4581**), and *SUPT3H-PTEN* fusion. His tumor was TMB-intermediate (6.39 Muts/Mb) and MS-stable. His PSA declined on pembrolizumab substantially from 2971 ng/mL to a nadir of 183 ng/mL. Of note, he was treated with both abiraterone and enzalutamide at separate points during his treatment course despite having previously progressed on both agents. Imaging after one year of pembrolizumab therapy revealed stable disease. He completed 18 cycles of pembrolizumab prior to being hospitalized for acute pancreatitis. His pancreatitis was thought to have been potentially caused by an auto-immune reaction to pembrolizumab; he was started on prednisone and received no further doses of pembrolizumab. At the time of treatment discontinuation his PSA reduction remained at approximately 80% from baseline.

Patent 3 is a 75-year-old Caucasian male, with initial Gleason 5+4, who developed disease progression after abiraterone, enzalutamide, sipuleucel-T, carboplatin, docetaxel, and cabazitaxel for mCRPC. He received treatment with combination enzalutamide and pembrolizumab despite having had previous progression on enzalutamide alone. His PSA declined substantially from 1048 ng/mL to 8.59 ng/mL within the first three months of therapy, with a nadir of 2.55. Imaging approximately three and a half months out revealed a partial response with decreased size of bulky retroperitoneal lymphadenopathy and retrocrural lymphadenopathy (**Figure 4**.) He completed 11 cycles of pembrolizumab prior to the study end date with his last PSA at 10.91 ng/mL. Molecular profiling was not available for the patient and thus the molecular association for his response remains unknown.

Patient 4 is 77-year-old Caucasian male, with initial Gleason of 3+4, who developed mCRPC progression despite prior abiraterone, enzalutamide, sipuleucel-T, radium-223, docetaxel, and cabazitaxel. He received treatment with combination enzalutamide and pembrolizumab despite having had previous progression on enzalutamide alone. His PSA declined rapidly from 63.66 ng/mL to a nadir of 3.96 ng/mL within the first three months of therapy. After the first four cycles of pembrolizumab, he was hospitalized for presumed immune mediated pneumonitis secondary to pembrolizumab and he was started on 60 mg prednisone daily with a slow taper. During this time his PSA increased back up to 11.62 ng/mL. Of note, it decreased back down to second nadir of 5.15 ng/mL after cessation of prednisone. He did not restart treatment with pembrolizumab prior to the study end date with his last PSA at 5.38 ng/mL. No evaluable imaging was available for comparison during the dates of this study.

Patient 8 is a 61-year-old Caucasian male, with initial Gleason of 4+5, who developed mCRPC progression despite abiraterone, enzalutamide, sipuleucel-T, docetaxel, cabazitaxel, and olaparib. His FoundationOne profile revealed an *ATM* (*Q2414**) mutation and an *LRP1b* (*R1815W*) mutation that was reported as a variable of undetermined significance (VUS). He received treatment with combination enzalutamide and pembrolizumab despite having had previous progression on enzalutamide alone. His PSA declined from 16.25 ng/mL to a nadir of 5.78 ng/mL with CT scan about two months into treatment showing stable disease. However, repeat CT scan almost five months out from treatment showed interval increase in size of an enhancing mass near the bladder concerning for metastatic disease and pembrolizumab was discontinued. He completed a total of 7 cycles prior to treatment discontinuation.

Of the remaining patients with genomic profiling available, only one other patient had a mutation in *LRP1b* (*LRP1b* loss of exons 4-91). He is a 76-year-old Caucasian male, initial Gleason unknown, who was previously treated with and progressed after standard ADT, abiraterone, enzalutamide, docetaxel, sipuleucel-T, radium-223, and cabazitaxel. His pretreatment scans showed widespread osseous disease along with bilateral adrenal nodules and multiple liver lesions. Nine days after his first dose of pembrolizumab he was found to have a platelet count of 21 x 10ˆ9/L after noticing spontaneous bruising under his eye. His baseline platelet count was around 150 x 10ˆ9/L and dropped to 112 x 10ˆ9/L about two weeks prior to his infusion, and to 64 x 10ˆ9/L on the day of his only pembrolizumab infusion. He was subsequently hospitalized after platelet counts continued to downtrend despite platelet transfusions and high dose steroids. During admission, a bone marrow biopsy was performed which revealed metastatic adenocarcinoma with extensive neoplastic involvement and minimal residual hematopoietic elements. After results of the biopsy, the patient elected to transition to home hospice. No post treatment PSA levels were available, and his thrombocytopenia was felt to be due to disease progression prior to starting pembrolizumab rather than an adverse event.

One patient had alterations in genes both for PD-L1 and for PD-L2, *CD274* amplification and *PDCD1LG2* amplification respectively. He is a 60-year-old Caucasian male, with initial Gleason 5+4, whose baseline PSA was low for his burden of disease at 0.44 ng/mL. The nadir during treatment was 0.39 ng/mL. He received a total of 9 cycles of pembrolizumab with stable PSA. However, staging scans at both three and six months showed progressive disease. Treatment was discontinued after six months scans showed increasing metastases in his lungs, liver, and pelvis.

Only one patient had a mutation in *CDK12* (*CDK12* splice site 2666+1G>T). He is an 82-year-old Caucasian male, with initial Gleason 4+5, who was previously treated with and progressed after standard ADT, abiraterone, enzalutamide, docetaxel, and cabazitaxel. His PSA was initially stable from 293 ng/mL to 285 ng/mL (checked at different laboratories); however, his PSA checked after his first four cycles showed increase to 417 ng/mL. CT scans at that time also showed interval progression of metastatic disease throughout the chest, abdomen, and pelvis along with new attenuating lesions in his femurs. He did not receive any further pembrolizumab infusions.
